# Challenges of and Solutions for Developing Tailored Video Interventions That Integrate Multiple Digital Assets to Promote Engagement and Improve Health Outcomes: Tutorial

**DOI:** 10.2196/21128

**Published:** 2021-03-23

**Authors:** Camilla Harshbarger, Olivia Burrus, Sivakumar Rangarajan, John Bollenbacher, Brittany Zulkiewicz, Rohit Verma, Carla A Galindo, Megan A Lewis

**Affiliations:** 1 Division of HIV/AIDS Prevention, Centers for Disease Control and Prevention Atlanta, GA United States; 2 Center for Communication Science, Research Triangle International Research Triangle Park, NC United States; 3 Public Health Division, SeKON Enterprise Inc Atlanta, GA United States; 4 Critical Focus Creative Durham, NC United States; 5 Office of the Associate Director for Communication, Centers for Disease Control and Prevention Atlanta, GA United States; 6 Translational Health Sciences Division, Research Triangle International Seattle, WA United States

**Keywords:** HIV video intervention, patient-provider communication, ART adherence, digital interventions, mobile interventions, computer based interventions, interactive technologies

## Abstract

**Background:**

Video is a versatile and popular medium for digital health interventions. As mobile device and app technology advances, it is likely that video-based interventions will become increasingly common. Although clinic waiting rooms are complex and busy environments, they offer the opportunity to facilitate engagement with video-based digital interventions as patients wait to see their providers. However, to increase efficiency in public health, leverage the scalability and low cost of implementing digital interventions, and keep up with rapidly advancing technology and user needs, more design and development guidance is needed for video-based tailored interventions.

**Objective:**

We provide a tutorial for digital intervention researchers and developers to efficiently design and develop video-based tailored digital health interventions. We describe the challenges and solutions encountered with Positive Health Check (PHC), a hybrid app used to deliver a brief, interactive, individually tailored video-based HIV behavioral counseling intervention. PHC uses video clips and multimedia digital assets to deliver intervention content, including interactive tailored messages and graphics, a repurposed animated video, and patient and provider handouts generated in real time by PHC.

**Methods:**

We chronicle multiple challenges and solutions for the following: (1) using video as a medium to enhance user engagement, (2) navigating the complexity of linking a database of video clips with other digital assets, and (3) identifying the main steps involved in building an app that will seamlessly deliver to users individually tailored messages, graphics, and handouts.

**Results:**

We leveraged video to enhance user engagement by featuring “video doctors,” full-screen video, storyboards, and streamlined scripts. We developed an approach to link the database of video clips with other digital assets through script coding and flow diagrams of algorithms to deliver a tailored user experience. We identified the steps to app development by using keyframes to design the integration of video and digital assets, using agile development methods to gather iterative feedback from multidisciplinary teams, and creating an intelligent data-driven back-end solution to tailor message delivery to individual users.

**Conclusions:**

Video-based digital health interventions will continue to play an important role in the future of HIV prevention and treatment, as well as other clinical health practices. However, facilitating the adoption of an HIV video intervention in HIV clinical settings is a work in progress. Our experience in designing and developing PHC presented unique challenges due to the extensive use of a large database of videos tailored individually to each user. Although PHC focuses on promoting the health and well-being of persons with HIV, the challenges and solutions presented in this tutorial are transferable to the design and development of video-based digital health interventions focused on other areas of health.

## Introduction

In this article, we present a tutorial on the challenges and solutions for designing and developing Positive Health Check (PHC), a brief and interactive digital video counseling intervention that can be used on computers or mobile devices (tablets, laptops) for persons with HIV who are engaged in clinical care. To improve health outcomes, the US public health community is leveraging the nation’s ubiquitous use of digital media [1]. Health interventions delivered through tablets, smartphones, and computers—including telehealth and wearable and remote monitoring devices—have been shown to improve health outcomes in multiple areas, including cancer prevention and control [2], alcohol intake and physical activity [3], sedentary behavior [4], and diabetes [5]. Given the ease of broad dissemination, digital interventions offer scalability and therefore the potential for greater population health impact. Scalability is a recent focus of HIV prevention efforts by the Centers for Disease Control and Prevention (CDC) through high-impact prevention [6], a strategy calling for effective, low-cost, and scalable HIV interventions, including those that can be disseminated via the internet [7].

The well-documented surge in the development of digital interventions for HIV prevention and care [8,9] aims to prevent the transmission of HIV and enhance the health of people with HIV through secondary prevention and management [10]. HIV digital interventions are appealing because of their ability to reach specific populations and social networks at high risk for HIV transmission [11-14], often through SMS text messaging [15] and social media [16]. Additionally, educational videos have been proven to improve knowledge, attitudes, and long-term behavior change in diverse populations [17], as seen in the precedent for implementing video interventions in sexually transmitted disease (STD) clinic environments [17-20] and other health care settings [2]. Through the implementation of video-based digital interventions in the waiting room or examination room, the clinic waiting time for patients with HIV can be used as a “teachable moment” [21].

Consequently, guidance is needed to help researchers and developers design, develop, and disseminate video-based digital interventions for HIV and other fields [2,22]. This tutorial addresses technical questions around the multiple challenges and solutions for the following: (1) using video as a medium to enhance user engagement with behavioral counseling interventions, (2) navigating the complexity of linking video with other digital assets through script coding and flow diagrams of tailoring algorithms, and (3) identifying main steps to building an app that will seamlessly deliver individually tailored messages, graphics, and handouts to users.

## Overview of PHC

### Theory and Rationale Underlying PHC

PHC’s primary aim is to enhance HIV viral suppression by providing individually tailored video messages on relevant topics, which are delivered by virtual doctors (played by actors) and mimic a clinical encounter. PHC’s secondary aims are to engage people with HIV in clinical care more regularly and to reduce HIV transmission risk. The theoretical foundations of PHC include the Information-Behavior-Motivation model [23], motivational interviewing [24], and the Transtheoretical model [25]. The PHC intervention design is based on similar video-based interventions that demonstrated efficacy for self-reported antiretroviral therapy (ART) adherence [26,27], reducing sex risk [28], and reducing viral load [27]. However, the scope of PHC goes beyond previous HIV video-based interventions in that it covers the above topics and also includes modules on treatment initiation, retention in medical care, safe injection drug use, and preventing mother-to-child transmission. The formative work that guided the development of PHC is described elsewhere [29].

PHC is also designed to strengthen patient-provider communication and empower patients to voice their concerns and ask questions in medical appointments. This supports current trends in patient education, where the patient and provider collaborate to make treatment decisions [30]. The video modules (such as adherence to medication) deliver tailored motivational messages, then users select questions to ask their provider and behavior change strategies. Behavior change strategies include, for example, creating reminders to take medications or getting tested for hepatitis C. These personal choices can make the intervention more motivating and increase engagement [31]. At the end of the intervention, the user receives a personalized handout documenting their selected questions and behavior change strategies. The handouts aim to strengthen the patient-provider relationship by encouraging providers to respond to the patient’s questions, which aligns with research that shows that patients may have better health outcomes when they feel their provider cares about them [32]. The behavior change strategies are for the patient to practice before the next clinic visit. In addition, the behavior change strategies are to be used as talking points to discuss with the provider, which gives the provider the opportunity to encourage positive behavior change, provide accountability, and give emotional support. Research shows that patients are more likely to sustain long-term behavior change if the patient makes an active commitment to do so [33]. Additionally, follow-up by the patient’s provider may signal trust in the intervention [31]. The rationale for this intervention structure is to provide a level of personal engagement with the tool that could lead patients from one stage of change to another (eg, from contemplating behavior change to taking action to change behavior).

### Rationale for a Video and Digital Platform

Multiple design considerations aimed to maximize PHC’s digital platform. First, we chose video for its visually engaging qualities and the ability to create a personal experience by tailoring the delivery of video clips to each individual user. The PHC video modules share information through visual, verbal, and written cues (on graphic overlays) and closed captioning. Second, the digital platform facilitates easy updates to PHC video clips and content in the supplemental resources. Third, video clips can be repurposed. For example, the animated condom video [34] featured in PHC that teaches how to use condoms correctly was repurposed from the video Safe in the City [20], an STD prevention intervention. Similarly, with permission, PHC video clips and the optional Extra Info microsite content featured at the end of the tool can be embedded in other interventions or used for discussion in face-to-face interventions (Multimedia Appendices 1-3). Fourth, the digital platform facilitated the use of a variety of digital assets, including tailored messages delivered via interactive video or graphics, the animated Safe in the City video, closed captioning, and user-generated patient/provider handouts. Fifth, we chose video in anticipation of meeting the demands of future users [2], as forecasts project that by 2022—along with accelerated use of Wi-Fi hotspots and increasing broadband speed—video will occupy about 84% of internet traffic [35].

### How PHC Architecture Works

PHC was designed to be used in the clinic setting, where staff provide earbuds and assign a tablet with a privacy screen to the patient. To use the intervention, patients log on to a device, answer four demographic tailoring questions, and then answer risk behavior assessment questions at the start of each intervention module. Based on this information, PHC generates tailored messages. A clip of the intervention that depicts an excerpt from the adherence module is available online [36].

PHC is interactive and allows the patient to choose their “video doctor” by race and gender; if desired, they can switch doctors at every use. In each session, the patient can select intervention content and drill down into the topics they wish to learn about. For example, under the broad topic of adherence, the patient can choose to learn about side effects of medication or taking ART and drinking alcohol. PHC delivers tailored messages to each patient, drawing from a database of 700 unique video clips. Additionally, the patient engages with interactive graphics to select from a menu of behavior change tips. To reach people with disabilities, the tool features closed captioning and keyboard navigation capabilities instead of a mouse or touchscreen. Extra Info, offered at the end of the tool, features peer-delivered educational videos and other resources.

Toward the end of the virtual clinic visit, PHC instantly generates two individually tailored handouts, a patient handout (Multimedia Appendices 4-5) and a summary handout for the provider, which the patient can elect to share or not. The patient can refer to the patient handout during the appointment and opt to send a PDF of it to their personal email account. As noted above, the handouts feature the patient’s self-selected behavior change strategies and questions for their provider.

## Challenges and Solutions to Designing and Developing PHC

### Overview

This section of the tutorial describes guidance on key solutions and challenges we faced in developing PHC around three topical areas related to user engagement, integration of intervention assets, and building the app to deliver complex tailored content (Table 1).

**Table 1:** Overview of challenges to and solutions for designing and developing Positive Health Check.

Challenge			Solution
**Topic 1. Enhancing user engagement through video design and production**			
	Leverage the medium of video to maximize user engagement		Limit distractions and make it look real Use entire video screen as a window into a virtual experience Streamline the scripts
	Plan for filming by visualizing the flow of scenes for the entire intervention		Develop storyboards that depict a series of images portraying actors’ positions on the screen along with their dialogue
**Topic 2. Linking videos and other digital assets through script coding and flow diagrams**			
	Linking the 700 videos to intervention scenarios while filming to ensure users’ tailored experience		Coding the shooting script to link to video clips
	Create a tool to monitor filming on the set		Create a master flow diagram of algorithms for each possible Positive Health Check user experience
**Topic 3. Building the Positive Health Check app**			
	**Programming the intervention tool**		
		How to plan to program the integration of video with multiple digital assets	Use keyframes to design the video screen by integrating video and other digital assets
		Test the integrity of the functional requirements used to design Positive Health Check	Use agile methods to gather rapid iterative feedback from a multidisciplinary team
		Deliver and display on-screen tailored content in real time as user inputs data	Build intelligent back-end design to support the delivery and display of users’ tailored content
	**Building a tool that performs efficiently on multiple devices**		
		Deliver a video-based intervention on mobile devices with limited resources, such as processor and memory (random access memory)	Use a single-page app that preloads common media/JavaScript files and avoid multiple page refreshes
		Use autoplaying of video clips and audio prompts on mobile devices	Develop hybrid apps that use device-specific settings to autoplay video
	**Finding a streaming video solution**		
		Selection of platform to ensure seamless delivery of sharp video with indiscernible buffering	Use streaming video from the Centers for Disease Control and Prevention web server
	**Planning for dissemination**		
		Ensure eventual dissemination and national-level scale-up to HIV clinics	Use of open web platform

### Topic 1: Enhancing User Engagement Through Video Design and Production

#### Overview

We recognized that several key components were needed to create a video tool that would bring about user engagement and lead patients to become more active in their own health. In this context, user engagement refers to “the micro-level of moment-to-moment engagement with the intervention” designed to promote behavior change to improve health outcomes [31].

#### Challenge: Leverage the Medium of Video to Maximize User Engagement

User engagement is a critical process to improve intervention effectiveness and behavior change [31]. Our challenge was to increase users’ engagement by limiting distractions, an important factor that informed creative decisions when designing PHC. Consequently, the visuals, location, acting style, wardrobe, dialogue, and camera movement all had to look real, be inviting, and impart confidence in the messages being received, without being distracting to the user. 

#### Solution: Limit Distractions and Make It Look Real

We approached this challenge to limit distractions by enhancing the “realness” of the virtual doctor visit. Although we considered using real-life doctors for the PHC, we ultimately decided to use actors because they are trained to deliver scripts verbatim from teleprompters in front of stage lights and crew. This ensured consistency of the message and delivery across the four virtual doctors embedded in the intervention. Actors appeared in clothing, hair, and makeup that would not reflect a specific decade and soon appear outdated. To mimic what a conversation might be like in a real doctor visit, instead of presenting all of the risk behavior assessment tailoring questions at the beginning of the tool, we embedded these questions throughout the experience. 

#### Solution: Use Entire Video Screen as a Window Into a Virtual Experience

We used a “corner to corner” 16 × 9 video screen that most closely approximates human natural visual perception, thus using the entire screen of the devices that would deliver the intervention. We used a video game structure to model high levels of engagement and immersion, treating each PHC screen depicted in the intervention as a “window” into a virtual experience. Finally, we overlaid graphics on top of the video so the user could still see the video image. This allows users to process the onscreen information but remain engaged and be rooted in the virtual doctor visit.

We also limited visual distractions by ensuring that the users’ focus stayed on the actor/doctor and their health messages, rather than on something in the background of the set. We accomplished this by filming in full high definition (1920 × 1080) on a Sony FS700 because the camera’s sensor size was large enough to provide a shallower depth of field, which would allow the background to be slightly blurred. 

#### Solution: Streamline the Scripts

Although the original scripts were developed collaboratively with end users and HIV providers [29], the creative director continued to translate these information-dense scripts into lines the actors could easily convey to PHC users and imbued them with more natural, conversational language. This resulted in a streamlined script to ensure end users would be more engaged and attentive.

#### Challenge: Plan for Filming by Visualizing the Flow of Scenes for the Entire Intervention

User inputs resulted in the delivery of tailored video clips and layered multimedia digital assets. Achieving these tailored responses required filming 168 separate video scenes for each of the four actors playing virtual doctors, which eventually would be programmed to play in multiple combinations. The challenge was to correctly plan for filming by visualizing the flow of scenes for each patient as if they were all one seamless interaction. 

#### Solution: Develop Storyboards That Depict a Series of Images Portraying the Actors’ Position on the Screen Along With Their Dialogue

To break down the complexity of PHC, we used storyboards to help visualize the look, feel, and flow of the tool. We created storyboards for every major PHC scenario. The storyboards were created by shooting still images of the location, then overlaying representations of the actor and graphics that would appear on screen. Those images were then paired with the relevant scripting (Multimedia Appendices 6-7). 

### Topic 2: Linking Videos and Other Digital Assets Through Script Coding and Flow Diagrams

#### Overview

Video is the basic medium of PHC, along with the other digital assets described above: interactive and tailored graphics, the repurposed Safe in the City animated video, closed captions, and user-generated handout templates. To make the intervention highly tailored and responsive to user input, we needed to connect scripted messages to a database of videos and digital assets so that when prompted by user input, the tool would correctly display these digital assets in congruity (or in sync) with the video.

#### Challenge: Linking the 700 Videos to Intervention Scenarios While Filming to Ensure Users’ Tailored Experience

PHC features 168 video clips for each of the four virtual doctors, and 28 clips common across doctors. It quickly became clear that all clips needed to be coded so we could efficiently coordinate the project. The challenge was to track the filming of all scenarios and ensure the correct use of all video clips according to programming algorithms. We needed a tool to coordinate the filming of all the clips and to ensure that when a user made selections while engaging with the intervention, PHC seamlessly delivered the appropriate video clip. 

#### Solution: Coding the Shooting Script to Link to Video Clips

The shooting script links video clips with tailored messages and user inputs—such as when the user selects topics for behavior change tips—and provides direction to the video director on how to compose each shot. The shooting script also described any graphics that would appear on the screen. Coding the videos based on their respective PHC intervention modules and creating a video catalog (codebook) from the shooting script standardized a naming convention for the 700 video clips. Consequently, the shooting script became the ultimate tool to link PHC videos to all intervention scenarios and user inputs.

#### Challenge: Create a Tool to Monitor Filming on the Set

PHC was designed as a highly tailored intervention with hundreds of different possible user pathways depending on user input. The challenge was to create a tool to plan for and monitor filming so that the team could see the logic and interconnection between all of the scenes and understand how the composition of each frame would inform the composition of subsequent video frames. 

#### Solution: Create a Master Flow Diagram of Algorithms for Each Possible PHC User Experience

**Figure 1:**
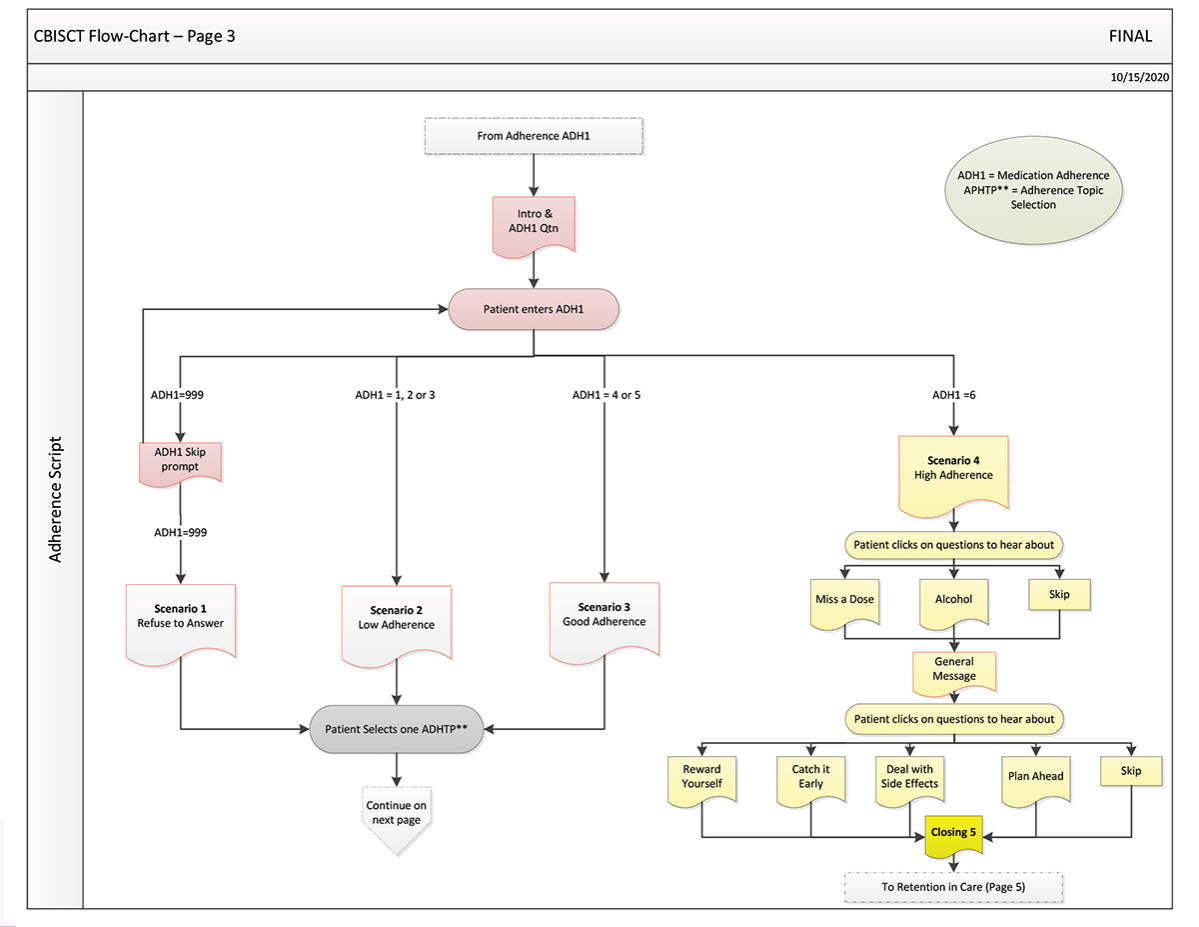
Positive Health Check flow diagram: adherence module pathway.

The solution to this challenge was to create the PHC flow diagram to show all the different tailoring paths a patient could take through the intervention. The flow diagram was used by the creative team to monitor the filming of the intervention and by the intervention programmer to guide the development of the algorithms underlying the intervention structure. This ensured that each scene would flow smoothly and be perceived as a natural progression in a conversation between a doctor and a patient. Figure 1 shows an example flow diagram for the adherence module.

### Topic 3: Building the PHC App

#### Overview

The PHC architecture achieves the following: (1) an intelligent, “back-end” design that refers to the underlying software framework (the app and the database) to provide tailored content based on user input, (2) a user-friendly interface that performs efficiently on multiple devices, such as computers, laptops, and tablets, and (3) a streaming video delivery solution that delivers high-quality video content.

The build-out started with the abovementioned coded shooting scripts and the tailoring flow diagram because these tools had applications for both filming and programming and were crucial linkage tools between the design and development teams at this stage. Building PHC involved finalizing technical functional requirements, prototyping, coding and programming, and performing internal quality testing (“sprints”). We also developed and tested each line of closed captioning that was paired with all script lines to ensure the captioning met Section 508 compliance requirements. Below, we describe the challenges and solutions encountered when building PHC, including programming the intervention tool, building a tool that performs efficiently on multiple devices, finding a streaming video solution, and planning for dissemination.

#### Programming the Intervention Tool

##### Challenge: How to Plan to Program the Integration of Video With Multiple Digital Assets

Each PHC module is constructed as a sequence of scenes appearing one after another, posing questions and imparting health messages. Each scene incorporates videos; input elements (such as check boxes) to collect user responses; display overlays to show topics and questions for patients to consider; and audio prompts to encourage patients to reengage during moments of inactivity, complete all questions, or avoid an early exit from the intervention. All the visual, HTML, and audio elements were choreographed in a scene to appear and animate on cue to provide the user with a visually rich and engaging experience. The challenge was how to create a tool to plan for, visualize, and document the placement of all these elements so that the videographer would frame each shot to give programmers the needed space for each element on the screen. 

##### Solution: Use Keyframes to Design the Video Screen by Integrating Video and Other Digital Assets

In film and video production, keyframes are pictures that define the timing and the starting and end points of transitions. Specifically, PHC keyframes in congruity with the scripts were the solution for defining the positioning and movement of the actors and displaying overlay images, check boxes, navigation buttons, and closed captioning. Keyframes proved to be a reliable tool for the graphic designers to communicate the intended look and user interface details to the developer who programmed the intervention. Multiple keyframes were created for each scene to show how visual elements would appear in the video at a specific point in time along with the narrative delivered by a doctor or nurse. Multimedia Appendix 8 shows an example of a keyframe for the adherence module, showing an actor/doctor in relation to the potential user and the graphic depicting choices of behavioral strategies designed to support HIV medication adherence, as well as navigation buttons.

##### Challenge: Test the Integrity of the Functional Requirements Used to Design PHC

The design of PHC had 200 functional requirements to guide the programming and delivery of 700 video clips to users. These functional requirements addressed the needs for specific intervention scenes, including the visual elements, data collected from or displayed to the patient, script to be conveyed through video, and all the probable user actions with corresponding responses from the system. Functional requirements also included rules to determine the next scene based on the user’s response to the current scene and/or all of the previous scenes. The challenge was to correctly develop, implement, and troubleshoot the functional requirements that would guide PHC programming and specify the interaction between user inputs and PHC outputs. 

##### Solution: Use Agile Methods to Gather Rapid Iterative Feedback From the Multidisciplinary Team and End Users

To address the challenge to verify and test the 200 functional requirements, we conducted numerous rapid and iterative feedback sessions or “sprints” in the application of “agile” methodology via collaborative, cross-functional, multidisciplinary teams [37,38]. As noted earlier, we also consulted end users, including patients and clinicians, across all phases of development [29]. This also entailed user testing of the prototypes and features during development. The solution was to rapidly develop, evaluate, and improve the PHC intervention tool to optimize and ready it for piloting. For example, in two sprints sessions, we resolved video buffering issues. To avoid buffering, we aimed to reduce video file size without increasing graininess or otherwise impacting the quality of the videos. After viewing several different resolutions, we selected an ideal file size to stream over a standard internet connection that would maintain video quality and eliminate buffering.

##### Challenge: Deliver and Display On-Screen Tailored Content in Real Time as the User Inputs Data

The individually tailored content distinguishes PHC from other digital interventions such as those delivered through text messaging. As noted previously, user engagement is critical to intervention effectiveness and behavior change. The challenge was to figure out how to design PHC to deliver and display tailored messages in real time along with all other tailored content from a large database of videos and other digital assets.

##### Solution: Build Intelligent Back-end Design to Support the Delivery and Display of Users’ Tailored Content

As noted earlier, the PHC back-end design refers to the underlying software and app, as well as a database with data sets that define the relationship between the scenes and videos, audio prompts, questions, and tips used to tailor PHC content to each user. This back-end design supports the delivery of individually tailored health messages based on user input. To achieve this end, our solution was to build an intelligent intervention engine in the back end that connects all app assets defined in data sets for a given scene and is fully data driven (ie, responsive to user input). It was built on the Microsoft ASP.NET MVC platform and an SQL Server database. The individual modules **(**such as adherence or retention in care) and the related scenes with video messages and graphical overlays are depicted in various configuration tables that reside in the SQL Server database.

#### Building a Tool That Performs Efficiently on Multiple Devices

##### Challenge: Deliver a Video-Based Intervention on Mobile Devices With Limited Resources, Such As Processor and Memory (Random Access Memory)

PHC was required to perform efficiently on mobile devices in spite of the challenge presented by the fact that, unlike desktop or laptop computers, mobile devices have limited memory and processing capabilities. These limiting features accommodate the small size of mobile devices and prevent overheating in the absence of fans and ventilation. However, the drawback is that these devices tend to crash when loading webpages with multiple media and code files. This is especially true when multiple full-page refreshes are needed during typical use of a website. In these mobile devices, memory and processing power are limited but valuable resources that need to be used efficiently to make intervention pages load and perform optimally.

##### Solution: Use a Single-Page App That Preloads Common Media/JavaScript Files and Avoid Multiple Page Refreshes

To address this challenge, PHC was developed as a single-page app that loads a webpage only once, at the beginning of the session; after that, the webpage content is manipulated through JavaScript programming. Additionally, the loading of required content is accomplished through asynchronous data transfers. The single-page app allows PHC to preload the video and JavaScript files, which are used across all or most of the intervention scenes during the initial page load. This approach also avoids full-page refreshes when transitioning from one scene to the next; instead, the scene-specific data, display overlays, and scripts are loaded asynchronously from the back end of the app based on the user inputs and intervention scenario into a screen area defined in the single-page app. The preloading of common videos and scripts into the tool significantly reduces load time for individual scenes. Additionally, the asynchronous loading of content into a single-page app decreases potential crashes by avoiding multiple-page refreshes involving videos, images, and JavaScript files.

##### Challenge: Use Autoplaying of Video Clips and Audio Prompts on Mobile Devices

Another key requirement for PHC is to provide an engaging intervention user experience by transitioning from one scene to the next without the need for the user to manually start and stop videos. All videos need to play automatically. PHC also has audio prompts to keep the user focused by encouraging them to answer questions and alerting them to any periods of inactivity. These audio prompts are programmed to automatically play in sync with all the video questions and messages, which worked flawlessly in computer web browsers. However, a problem occurred with the autoplaying of videos on Apple and Android tablets. These mobile operating system vendors restrict video autoplay within their web browsers, such as Safari or Chrome. These restrictions prevent unexpected user data charges, which can be particularly expensive for cellular internet connections. Consequently, the challenge was to figure out how to play PHC videos on mobile devices. 

##### Solution: Develop Hybrid Apps That Use Device-Specific Settings to Autoplay Video

To address this issue, we developed hybrid apps for Apple iPads and Android tablets. Hybrid apps are web apps wrapped inside native apps that are deployed in mobile app stores, such as Google Play or Apple Store. This hybrid approach allowed for the autoplay of PHC videos on these devices. In addition, the PHC hybrid app will allow for continual enhancements to PHC and modification of the user experience. Most importantly, by using this solution, we can deploy changes to PHC through the web without needing to update and deploy mobile apps each time there is an update to PHC. 

#### Finding a Streaming Video Solution

##### Challenge: Selection of a Platform to Ensure Seamless Delivery of Sharp Video With Indiscernible Buffering

This challenge involved identifying a server that would efficiently deliver video content to PHC users. Cost, flexibility, data security, and streaming speed were important considerations for the final solution. YouTube provides a low-cost streaming option but offers little flexibility when it comes to controlling and displaying content. Videos hosted by YouTube, for example, cannot be played directly through the PHC app’s HTML5 player, but instead must use Google’s YouTube player. Additionally, the YouTube player always includes Google’s advertisements and usage tracking code. As an alternate streaming solution, we considered Akamai’s cloud delivery platform, which uses caching to rapidly stream content from servers globally. However, Akamai pricing was prohibitive.

##### Solution: Use Streaming Video From the CDC Web Server

After researching the above potential solutions to this challenge, we decided to implement a streaming video solution by hosting video content on a CDC internal web server. This decision was confirmed after evaluating video performance on delivering video content rapidly and securely using load testing with many concurrent users. The videos were fine-tuned to be of good quality yet lower file size to support delivery over standard internet connections. Although this approach is adequate for use within clinical settings, it may not scale well as the number of concurrent users increases. We recommend the use of cloud-based content delivery networks to stream videos for wider dissemination.

#### Planning for Dissemination

##### Challenge: Ensure Eventual Dissemination and National-Level Scale-up to HIV Clinics

We designed and developed PHC to create conditions for greater population impact, and decisions were made with the goal of disseminating PHC to HIV clinics, if it was found to be effective. We aimed to build an intervention that could be feasibly implemented in busy HIV clinic environments and would be relevant to clinic providers and patients with HIV engaged in clinical care. The challenge was to design an intervention tool that will be available and accessible on multiple commonly used devices.

##### Solution: Use of Open Web Platform

We achieved a solution for possible future compatibility by developing PHC using tools and technologies supporting the World Wide Web Consortium’s open web standards. Apps using the open web platform are scalable, cross-platform compliant, and supported by multiple browsers without sacrificing user experience [39]. Standards-based systems are nonproprietary and include, for example, HTML5 and JavaScript. The front end of PHC is built on these open web standards–based tools. All the graphical interfaces are developed using HTML enriched for an interactive mobile experience using well-designed graphical images. This is preferable to creating a solution using third-party commercial off-the-shelf tools, such as Adobe Flash (Adobe), and enables PHC to run on any standard web browser without the need to install additional software tools.

## Conclusion

Video-based digital health interventions will continue to play an important role in the future of HIV care and other clinical health practices. This is, in part, due to the ever-increasing popularity of video as a way to consume information across all sectors of society and multiple disciplines, including medicine [40,41]. Notably, the condom use animated video clip from Safe in the City (now repurposed for PHC) has received 980,000 views on YouTube. However, facilitating the adoption of an HIV video intervention in HIV clinical settings is still a work in progress. It remains a challenge to build individually tailored interventions that engage users, keep interventions up to date, and implement and scale up interventions in busy clinic workflows. We chose to design a tailored video-based intervention with the hope that it could meet these challenges and benefit the health of patients with HIV engaged in clinical care and improve clinics’ performance metrics around viral suppression and retention in care. We believe that the return on investment of developing this highly interactive and tailored intervention can be considered beneficial if the intervention is shown to be effective and scaled up and implemented at a low cost by HIV clinics. We are currently conducting a pragmatic type 1 hybrid trial [42] to test the effectiveness of PHC in improving health outcomes for patients with HIV engaged in clinical care, as well as to test the feasibility of implementing PHC in HIV clinics. The study also includes collecting data on the costs associated with implementing PHC in clinical settings [43].

Our experience in designing and developing PHC presented unique challenges because of the extensive use of a large database of videos individually tailored to each user, and also revealed other challenges that are well known to investigators who seek to develop digital interventions [44]. We believe that researchers and practitioners who seek to support and change health behavior using digital technology can benefit from our learnings, because the speed by which digital health interventions capitalize on technological advances far outpaces the development, testing, and deployment of health behavior change interventions. The tutorial presented here on the complex challenges and solutions involved in designing and developing PHC aims to support researchers in HIV and beyond who wish to keep pace with approaches to and the technology behind video-based digital interventions [37,38].

## Appendix

Multimedia Appendix 1 Positive Health Check: Extra Info.

Multimedia Appendix 2 Positive Health Check: Extra Info landing page.

Multimedia Appendix 3 Positive Health Check: Your Sexual Relationships.

Multimedia Appendix 4 Positive Health Check patient handout (image 1).

Multimedia Appendix 5 Positive Health Check patient handout (image 2).

Multimedia Appendix 6 Excerpt of Positive Health Check storyboard: opening script (image 1).

Multimedia Appendix 7 Excerpt of Positive Health Check storyboard: opening script (image 2).

Multimedia Appendix 8 Positive Health Check keyframe for the adherence module (image 2).

## Abbreviations

ART: antiretroviral therapy
CDC: Centers for Disease Control and Prevention
PHC: Positive Health Check
STD: sexually transmitted disease
