# Supplementary material for: Challenges of and Solutions for Developing Tailored Video Interventions That Integrate Multiple Digital Assets to Promote Engagement and Improve Health Outcomes: Tutorial
Source: JMIR Mhealth Uhealth. 2021 Mar 23;9(3):e21128. doi: 10.2196/21128 (PMC8294466; doi:10.2196/21128)
Supplement: Multimedia Appendix 1 [file mhealth_v9i3e21128_app1.docx]

**Multimedia Appendix 1**

**Positive Health Check Extra Info**

Positive Health Check (PHC) Extra Info is a secure website that provides a wealth of information relevant to persons with HIV. PHC study participants are provided access to the website after each visit to the PHC intervention and can access it at any time with their unique PHC credentials. The website was designed to supplement patients’ HIV primary care visits by empowering and supporting patients in the management of their overall health given their HIV diagnosis.

Extra Info includes 11 sections with HIV-related topic areas ranging from “What you need to know about HIV” to “Mental Health” and “Insurance and Financial Resources” (Figure A-1). Within each section, the patient learns more about the featured topic area by navagating to credible external websites, factsheets, and locator services, and by viewing videos of people with HIV sharing testimonials with helpful tips, encouragement, and peer support (Figure A-2).

Extra Info was designed to serve as a patient-driven resource, accessed at the PHC user’s convenience from a device at their clinic or from a personal device at home. The Extra Info website was designed to be easy to use with a simple and familiar menu-driven user interface and is monitored to ensure resources such as external links continue to be available and that they are up to date. To gauge patients’ exposure to and use of this resource, the website was programmed to capture certain metrics, such as when and how many times patients accessed Extra Info, whether it was accessed in the clinic or offsite, and for what length of time it was accessed.

The screen shots below show the Extra Info landing page (Figure A-1) and the page about healthy sexual relationships with embedded videos from the Positive Project now housed at the University of California, San Francisco Center for AIDS Prevention Studies (Figure A-2).

**Figure legends:**

Figure. A-1. Positive Health Check: Extra Info landing page

Figure A-2. Positive Health Check: Your Sexual Relationships
