# Supplementary figures and images for: Challenges of and Solutions for Developing Tailored Video Interventions That Integrate Multiple Digital Assets to Promote Engagement and Improve Health Outcomes: Tutorial
Source: JMIR Mhealth Uhealth. 2021 Mar 23;9(3):e21128. doi: 10.2196/21128 (PMC8294466; doi:10.2196/21128)

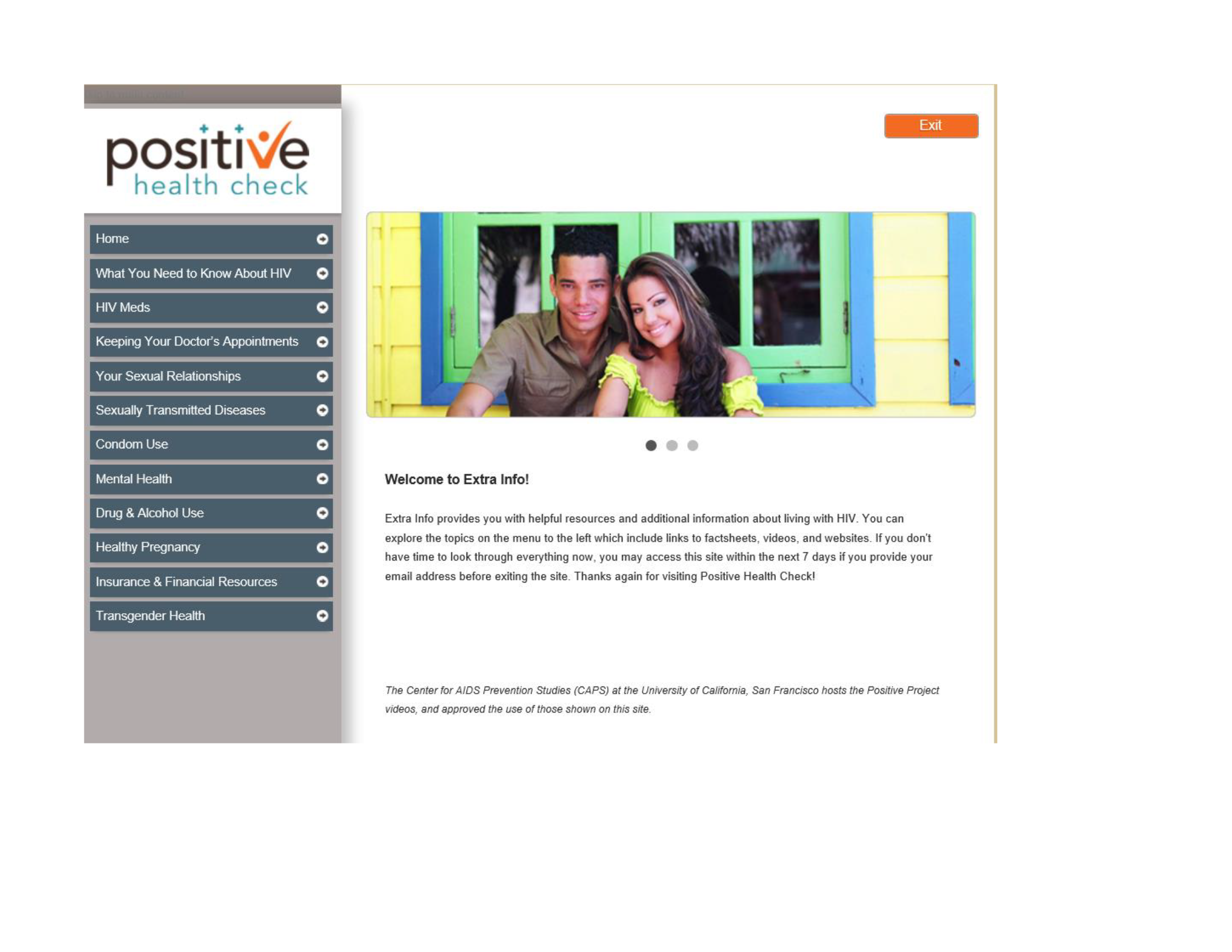

Supplement: Multimedia Appendix 2 [file mhealth_v9i3e21128_app2.png]

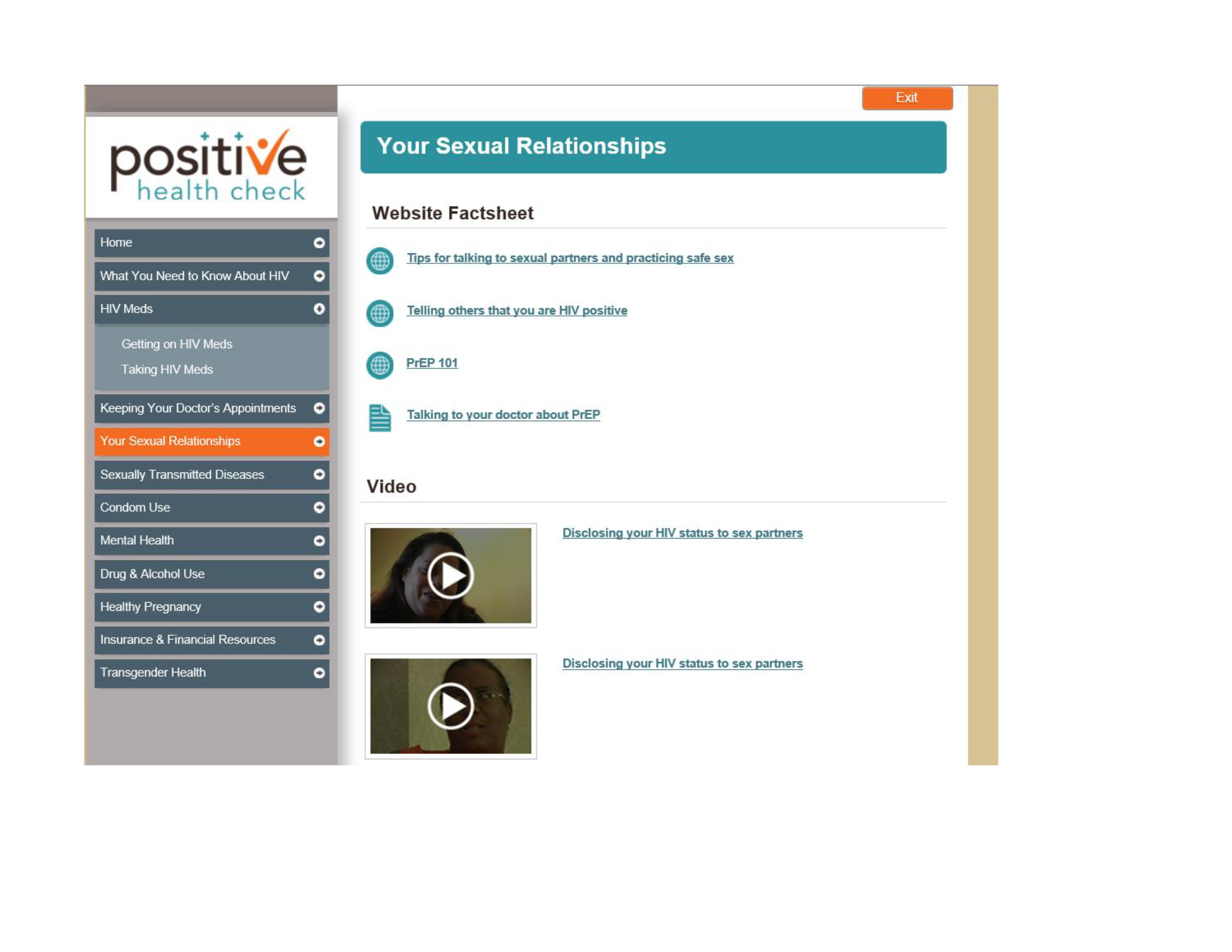

Supplement: Multimedia Appendix 3 [file mhealth_v9i3e21128_app3.png]

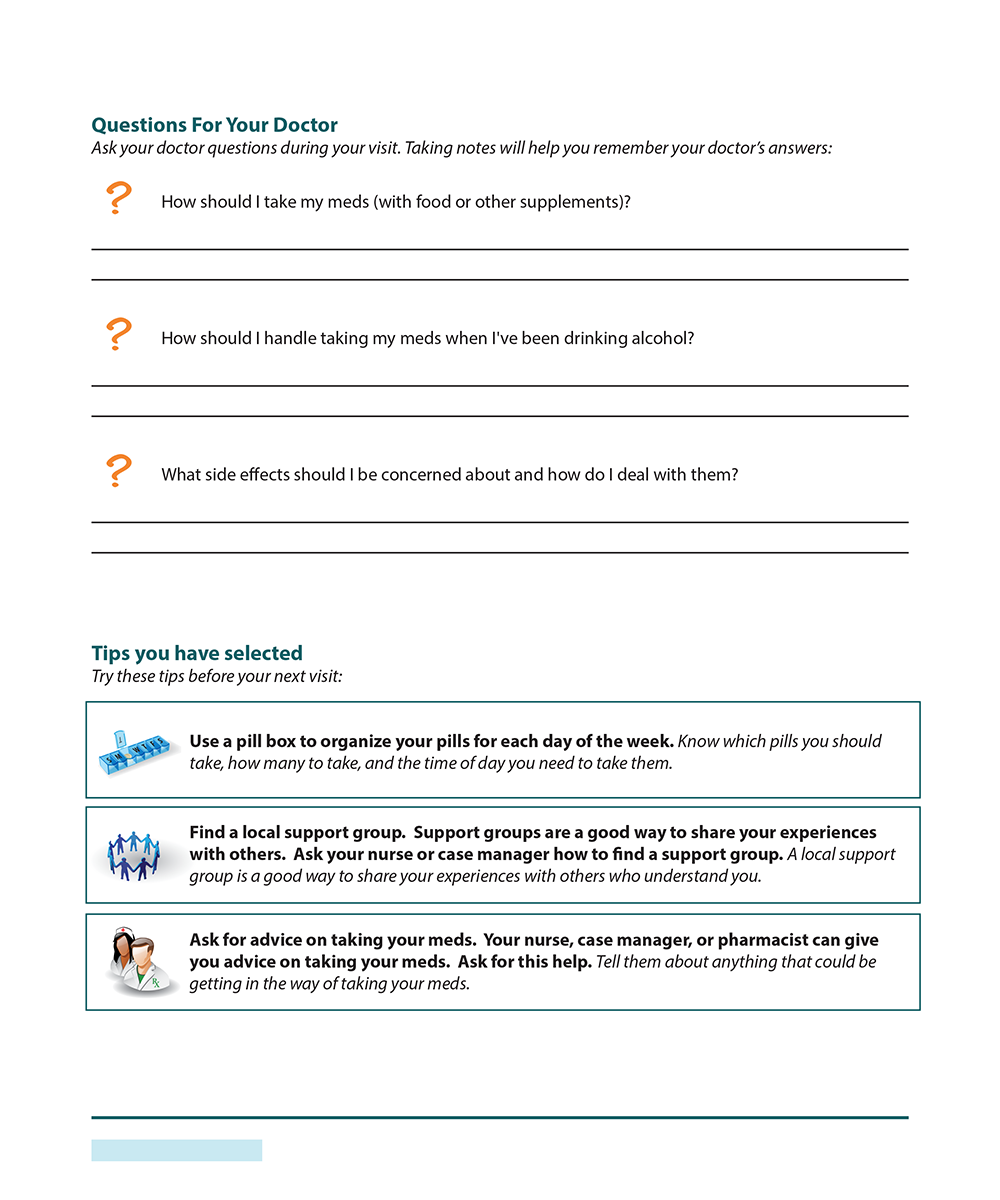

Supplement: Multimedia Appendix 4 [file mhealth_v9i3e21128_app4.png]

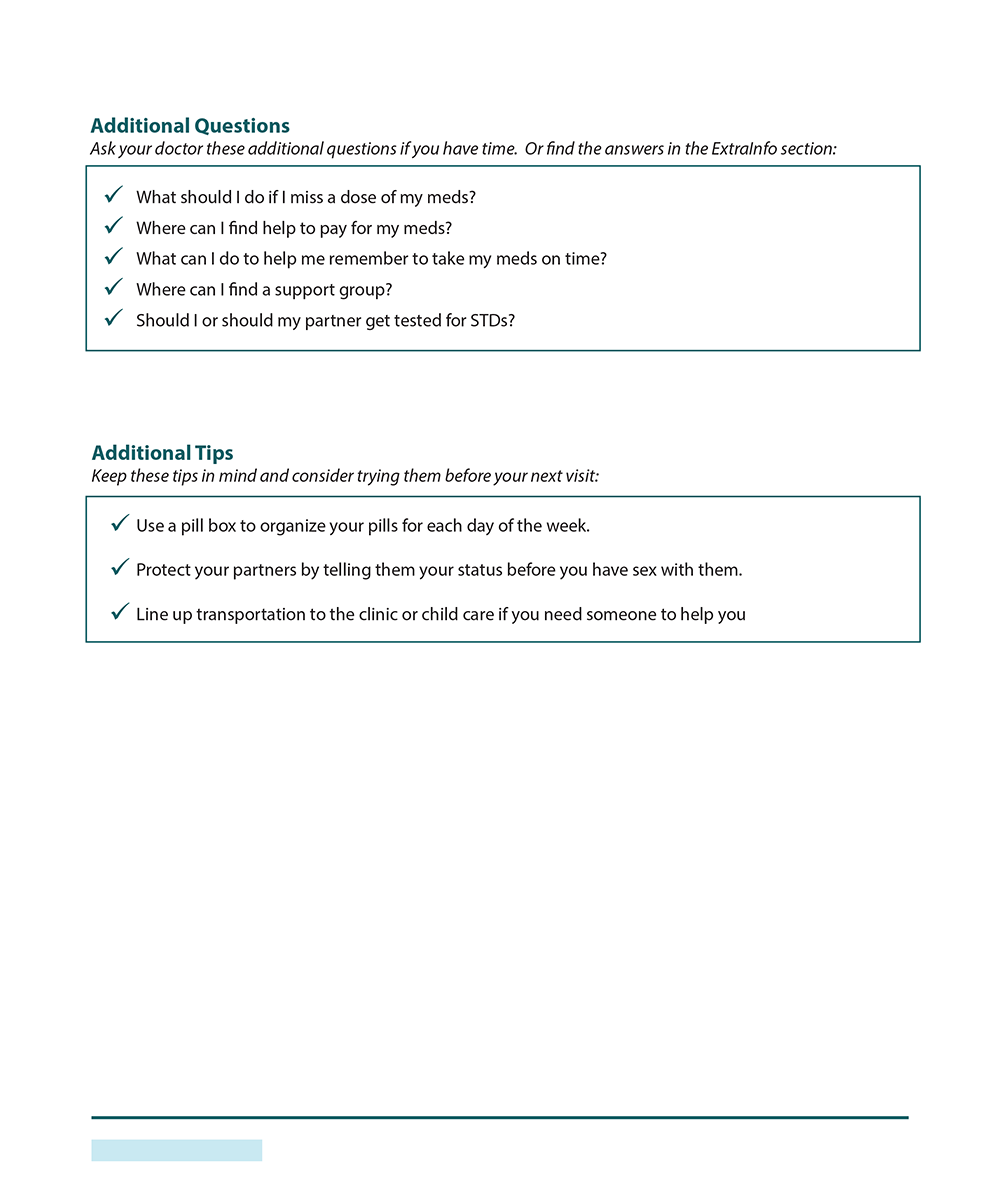

Supplement: Multimedia Appendix 5 [file mhealth_v9i3e21128_app5.png]

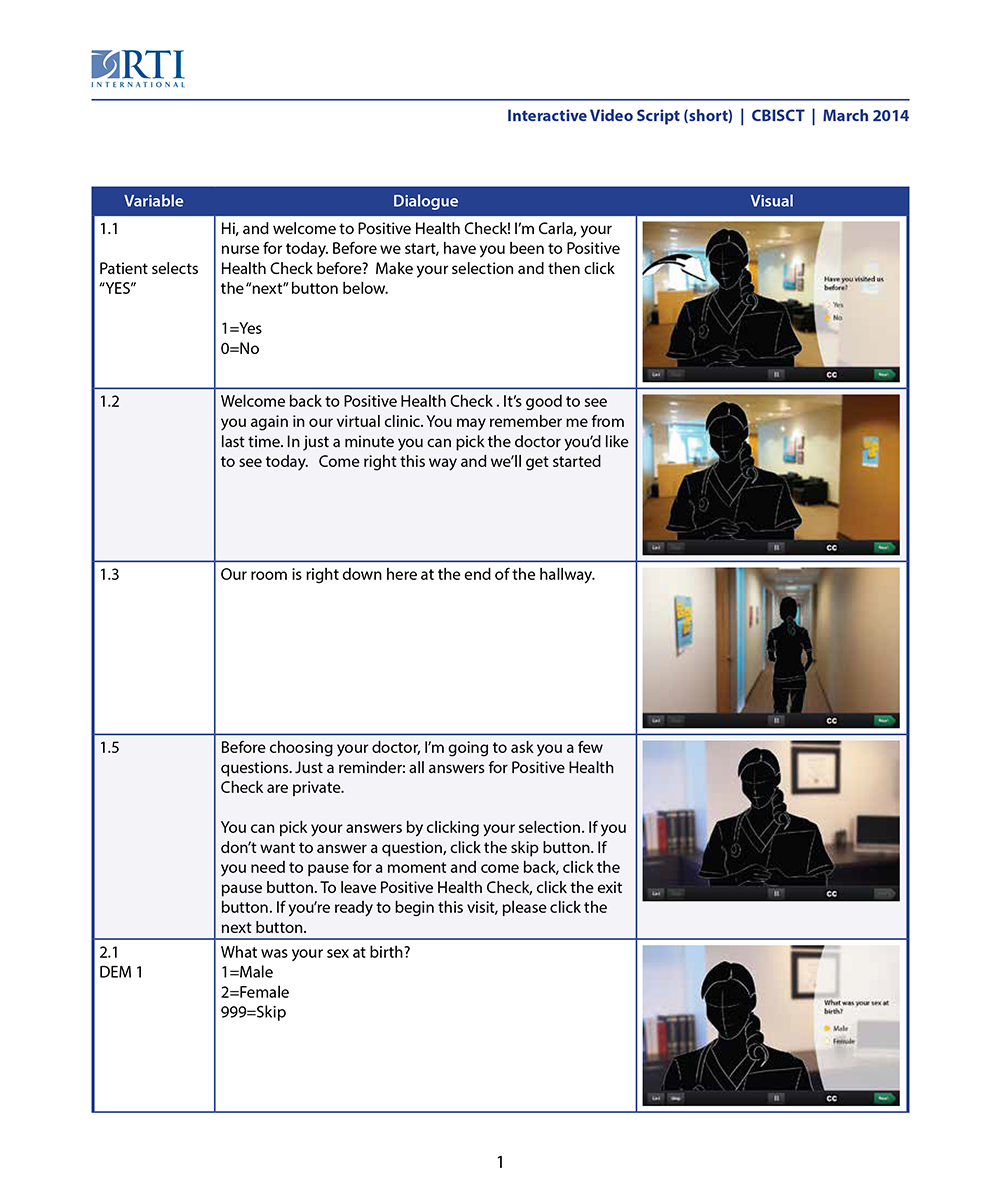

Supplement: Multimedia Appendix 6 [file mhealth_v9i3e21128_app6.png]

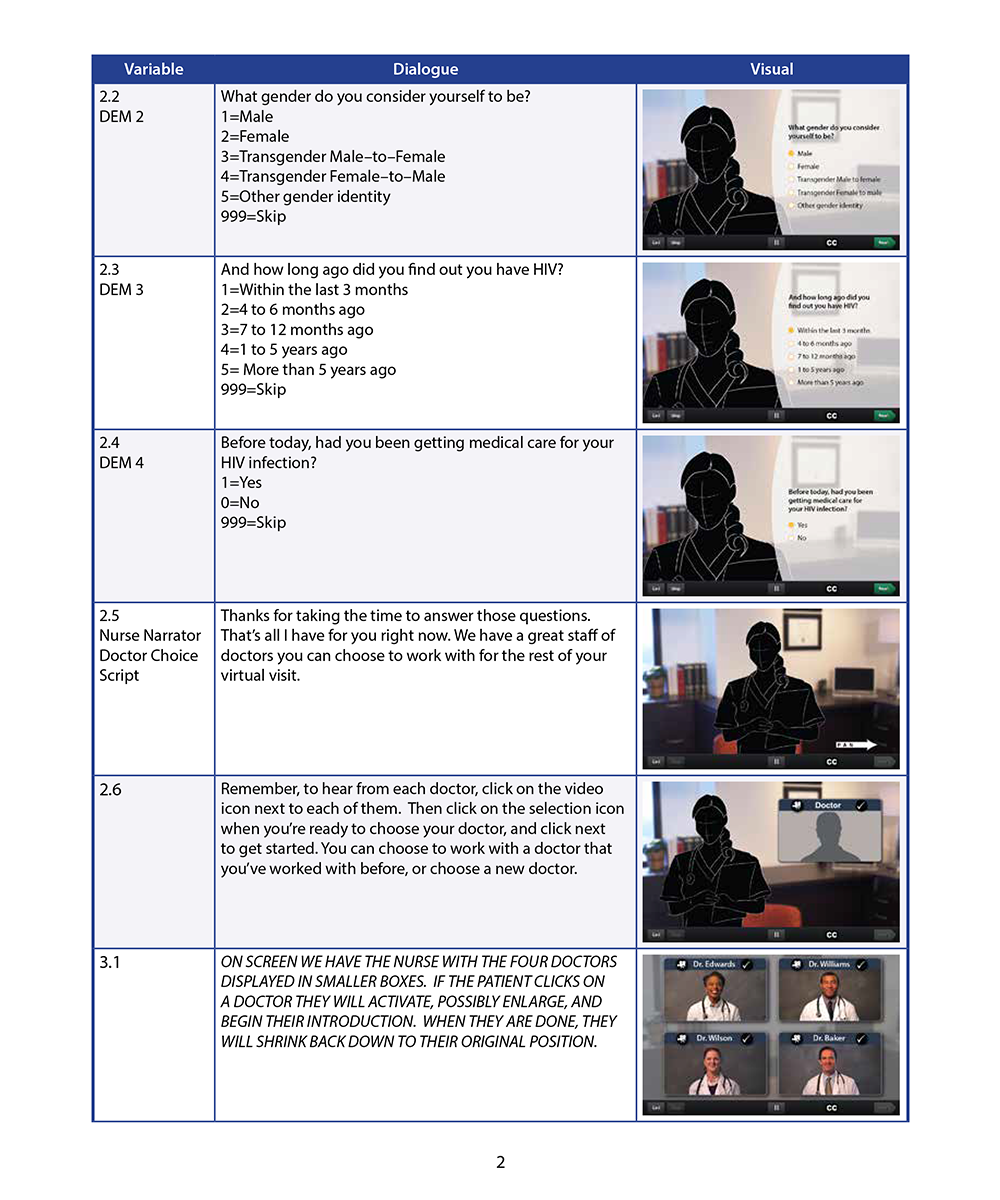

Supplement: Multimedia Appendix 7 [file mhealth_v9i3e21128_app7.png]

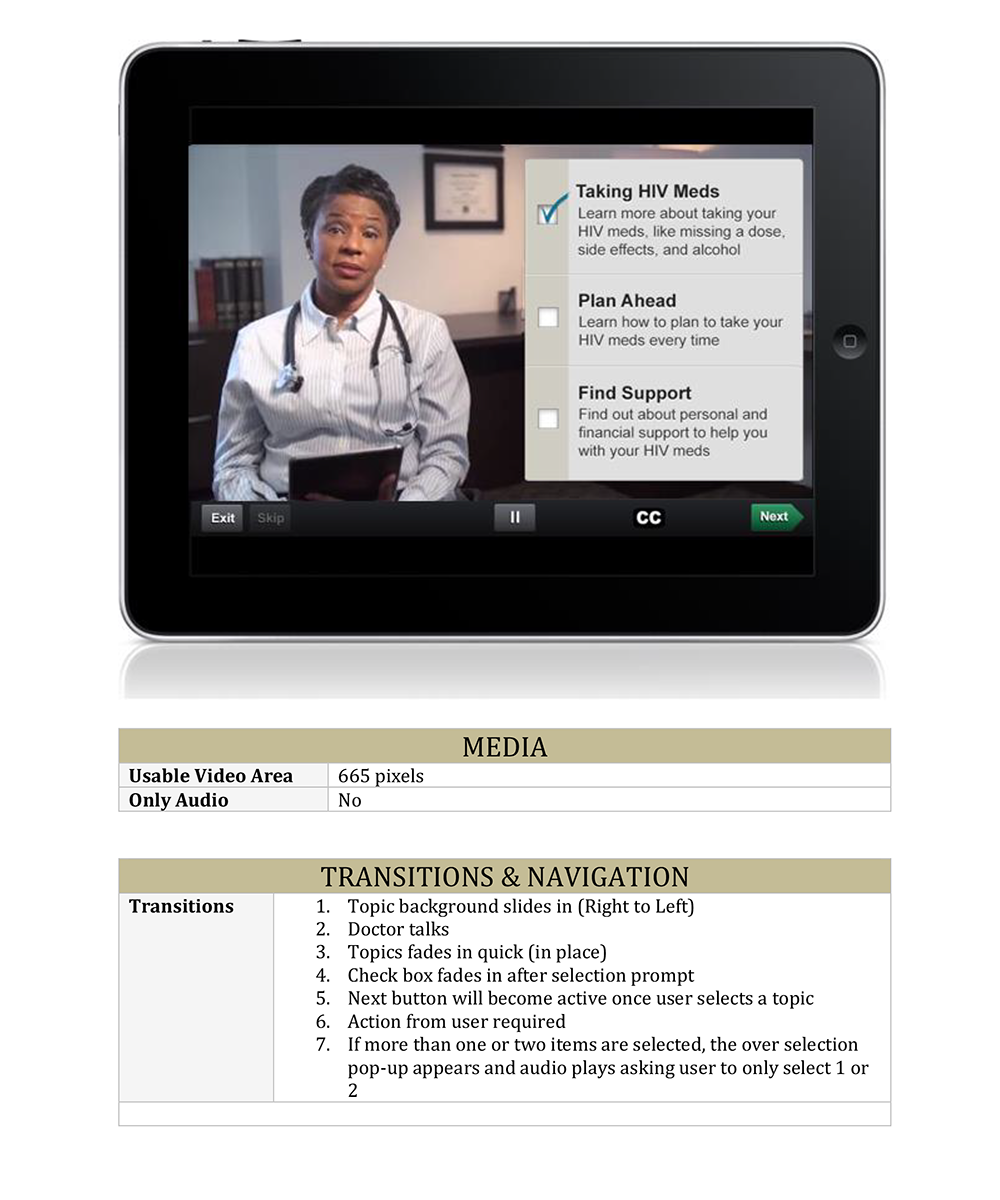

Supplement: Multimedia Appendix 8 [file mhealth_v9i3e21128_app8.png]
